# Supplementary material for: Expanding the Phenotype of Hereditary Congenital Facial Paresis Type 3
Source: Int J Mol Sci. 2023 Dec 21;25(1):129. doi: 10.3390/ijms25010129 (PMC10779017; doi:10.3390/ijms25010129)
Supplement: Supplementary file 1 [file ijms-25-00129-s001.zip › Figure S1.pdf]

Figure S1: Paternity testing using microsatellite markers. Different colors indicate different short tandem repeats.

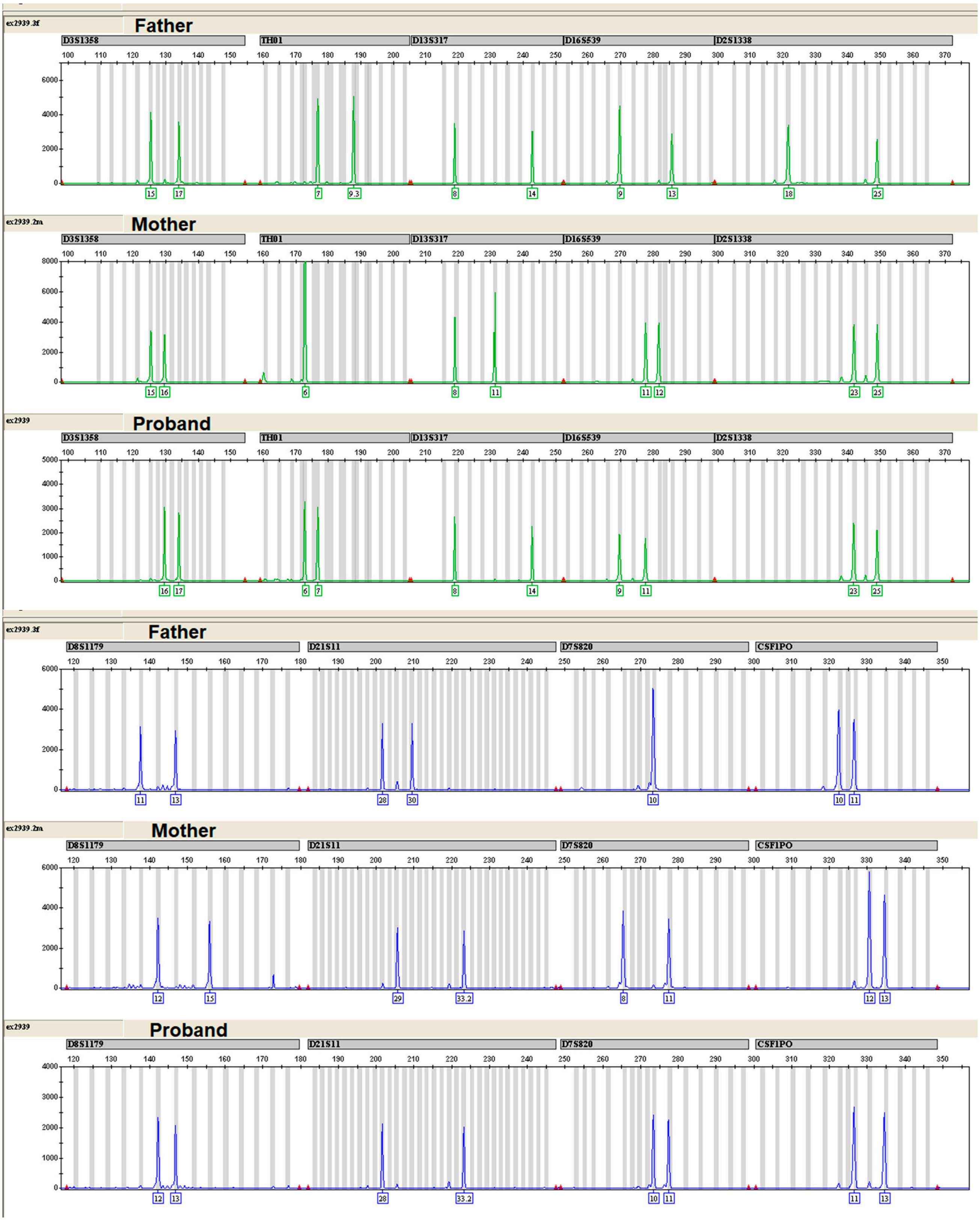

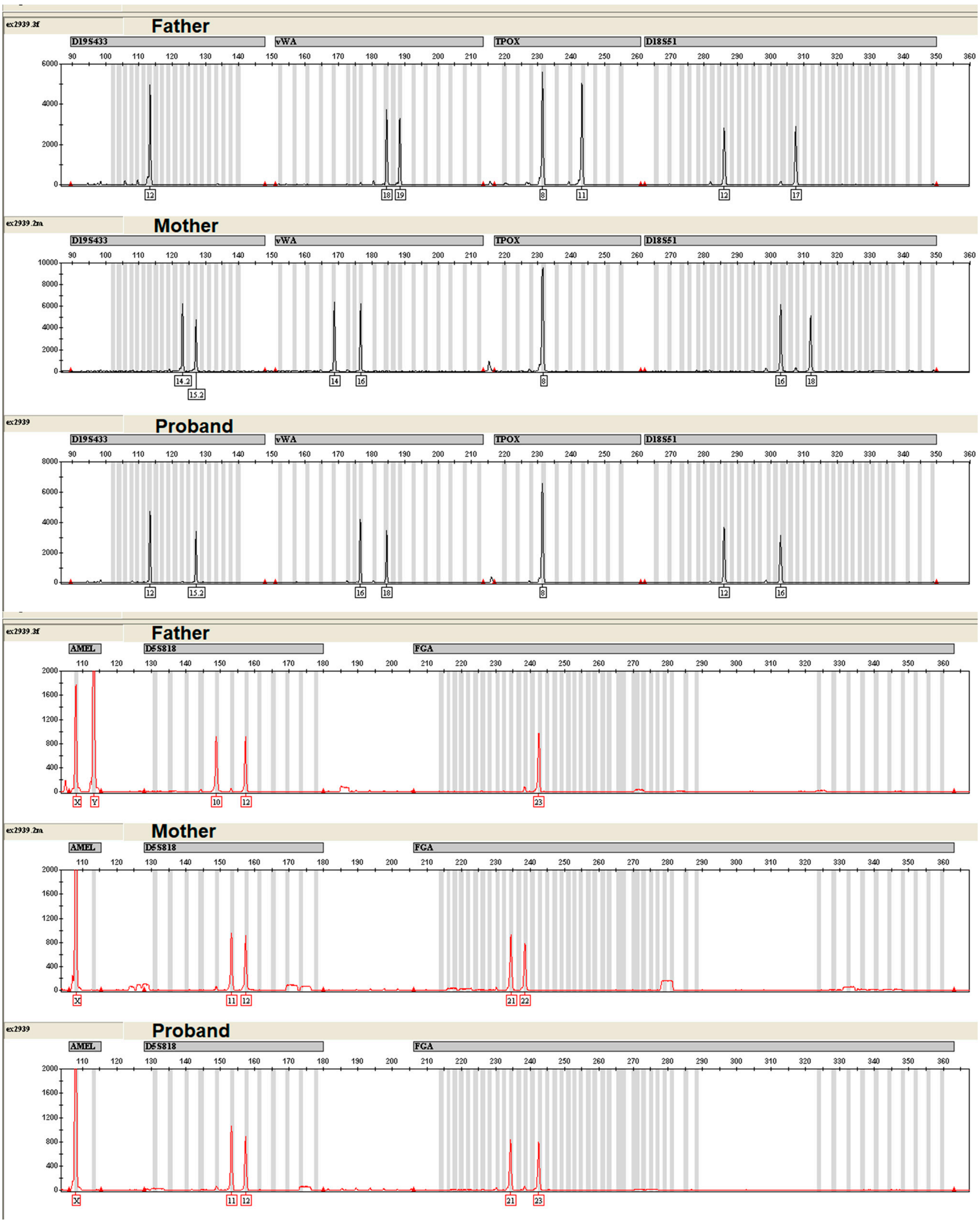
ex2939.3f
Father
D19S433
vWA
TPOX
D18S51
12
18 19
8
11
12
17
ex2939.2m
Mother
D19S433
vWA
TPOX
D18S51
14.2
15.2
14
16
8
16
18
ex2939
Proband
D19S433
vWA
TPOX
D18S51
12
15.2
16
18
8
12
16
ex2939.3f
Father
AMEL
D5S818
FGA
X
Y
10
12
23
ex2939.2m
Mother
AMEL
D5S818
FGA
X
11
12
21
22
ex2939
Proband
AMEL
D5S818
FGA
X
11
12
21
23
